# Technological Capabilities to Assess Digital Excellence in Hospitals in High Performing Health Care Systems: International eDelphi Exercise

**DOI:** 10.2196/17022

**Published:** 2020-08-18

**Authors:** Marta Krasuska, Robin Williams, Aziz Sheikh, Bryony Dean Franklin, Catherine Heeney, Wendy Lane, Hajar Mozaffar, Kathy Mason, Sally Eason, Susan Hinder, Rachel Dunscombe, Henry W W Potts, Kathrin Cresswell

**Affiliations:** 1 Usher Institute University of Edinburgh Edinburgh United Kingdom; 2 Institute for the Study of Science, Technology and Innovation University of Edinburgh Edinburgh United Kingdom; 3 UCL School of Pharmacy London United Kingdom; 4 National Institute for Health Research Imperial Patient Safety Translational Research Centre London United Kingdom; 5 National Health Services Arden and Greater East Midlands Commissioning Support Unit Warwick United Kingdom; 6 Business School University of Edinburgh Edinburgh United Kingdom; 7 Imperial College London United Kingdom; 8 KLAS Research’s Arch Collaborative London United Kingdom; 9 Institute of Health Informatics University College London London United Kingdom

**Keywords:** digital excellence, digital maturity, Delphi technique, hospitals, eHealth

## Abstract

**Background:**

Hospitals worldwide are developing ambitious digital transformation programs as part of broader efforts to create digitally advanced health care systems. However, there is as yet no consensus on how best to characterize and assess digital excellence in hospitals.

**Objective:**

Our aim was to develop an international agreement on a defined set of technological capabilities to assess digital excellence in hospitals.

**Methods:**

We conducted a two-stage international modified electronic Delphi (eDelphi) consensus-building exercise, which included a qualitative analysis of free-text responses. In total, 31 international health informatics experts participated, representing clinical, academic, public, and vendor organizations.

**Results:**

We identified 35 technological capabilities that indicate digital excellence in hospitals. These are divided into two categories: (a) capabilities within a hospital (n=20) and (b) capabilities enabling communication with other parts of the health and social care system, and with patients and carers (n=15). The analysis of free-text responses pointed to the importance of nontechnological aspects of digitally enabled change, including social and organizational factors. Examples included an institutional culture characterized by a willingness to transform established ways of working and openness to risk-taking. The availability of a range of skills within digitization teams, including technological, project management and business expertise, and availability of resources to support hospital staff, were also highlighted.

**Conclusions:**

We have identified a set of criteria for assessing digital excellence in hospitals. Our findings highlight the need to broaden the focus from technical functionalities to wider digital transformation capabilities.

## Introduction

It is now widely recognized that health information technology (HIT) has significant potential to transform health care systems and support continuous quality improvement efforts [1]. With growing recognition of this potential has come a strong international drive towards creating digitally advanced health care organizations. To this end, hospitals worldwide are now implementing ambitious digital transformation programs [2,3].

There are various ways to conceptualize and measure digital excellence in health care [4,5]. These approaches vary in scope from highly specialized models, focusing on a specific technological subsystem [6] to those assessing digital transformation across an entire hospital, and others encompassing the wider integrated health and care ecosystem [7]. The origin of these models is also diverse, including international health care industry organizations such as the Healthcare Information and Management System Society (HIMSS) Analytics [8], national health care providers [9], and academic groups [10]. Common to all existing frameworks is the concept of digital transformation progressing towards advanced levels of digital maturity through a defined set of stages associated with different technological capabilities. Perhaps the best known of these is the HIMSS Analytics Electronic Medical Record Adoption Model (HIMSS EMRAM; Textbox 1). Policymakers and health care organizations commonly use these frameworks for baseline assessments of current levels of digital maturity and as a roadmap for a desired future state of maturity. As such, these frameworks actively shape the direction of digital transformation [11].

Despite substantive worldwide efforts to promote digital excellence, there is no consensus on how to conceptualize it, what capabilities characterize a digitally excellent hospital, and how to best measure progress in a changing environment [12]. New models are beginning to emerge that acknowledge the importance of locally formed priorities and the changing nature of what constitutes digital excellence over time. In this study, we sought to identify and reach consensus on a defined set of internationally relevant technological capabilities for hospitals in order to address current gaps in approaches to conceptualizing and assessing digital excellence.

**HIMSS Analytics Electronic Medical Record Adoption Model (EMRAM).**

The HIMSS EMRAM classification evaluates the extent to which electronic medical records (EMRs) have been adopted within a hospital over eight progressive stages (Levels 0-7).

A hospital’s digital transformation begins at Level 0, in which no electronic laboratory, pharmacy, or radiology systems are installed. The hospital then moves through Levels 1-7 by progressive adoption of various aspects of EMRs. These include limited ancillary departmental systems (Level 1), and adoption across an increasing number of hospital departments (Levels 1-6), culminating in a virtually paperless environment with complex EMRs implemented in over 90% of the hospital’s departments (Level 7).

A hospital can be assessed against the HIMSS classification to establish its current HIMSS Level, which in turn highlights what further technological capabilities the hospital needs to reach the next level of the HIMSS classification. HIMSS Level 7 is often considered a ‘gold standard’ for the digitization of hospitals and an aspirational endpoint guiding the design of a hospital’s digital strategy.

## Methods

### Study background

This work was conducted as part of a national evaluation of the National Health Service (NHS) Global Digital Exemplar (GDE) Programme in England [13]. The GDE Programme aims to create a cohort of digitally excellent hospitals (“Exemplars”), which are expected to share their experiences and learning with other health care providers and contribute toward creating a national learning health care ecosystem [3]. We followed the reporting recommendations for Delphi studies outlined by Boulkedid and colleagues [14].

### Overview of the Delphi Method

The Delphi technique is a structured process that involves presenting a series of surveys to a group of experts to seek their agreement on statements relating to a particular issue [15]. An initial survey informs the development of a second survey, which is returned to the experts, who are asked to reconsider their initial judgment in light of feedback from the first round. Consecutive rounds are carried out until consensus is reached [16]. The key strength of the Delphi method is that it supports consensus development in an area of uncertainty or limited empirical evidence [17]. The method allows drawing on a wide range of experts’ knowledge and experiences. The feedback offered to participating experts between rounds has the potential to widen participants’ outlooks and stimulate new ideas that can be expressed in subsequent rounds [17]. The anonymity offered by the method (the identity of experts and their contributions are not known to other experts taking part in the Delphi exercise or the public) also has potential to facilitate disclosing opinions that may be underrepresented or not expressed in other forms of consensus-building approaches where participants are aware of each other’s identity. The potential risks associated with the Delphi approach include lack of accountability for anonymously presented views, and risks generally associated with consensus-building approaches such as group-think and lack of diversity of views represented in the outcomes [17]. After considering its strengths and weaknesses, we decided that the Delphi method would be an appropriate approach for addressing our aim of developing international agreement on a defined set of technological capabilities to assess digital excellence in hospitals in high performing health care systems.

To ensure a reasonable geographical spread of experts and relatively prompt completion of the Delphi exercise, we used a modified Delphi approach utilizing electronic communication with experts [18]. The modified electronic Delphi (eDelphi) technique has been widely used in health care and medical informatics, for example, to establish a set of readiness criteria for HIT innovations [19], to define key performance indicators to benchmark hospital information systems [20], and to identify ways to improve the delivery of medication alerts within computerized physician order entry (CPOE) systems [21].

The study took place between July 2018 and January 2019. Ethics approval was obtained from an Institutional Review Board at the School of Social and Political Science at The University of Edinburgh, United Kingdom. The Qualtrics platform was used to develop an online survey and collect data. SPSS Version 24 was used to conduct quantitative analyses, and NVivo Version 12 was used to analyze free-text responses.

### The eDelphi Process

#### Identification of Experts

We identified a diverse group of international experts in the field of health informatics from leading clinical, academic, public, and vendor organizations, aiming for maximum variation in terms of geographical location, background (eg, academic, clinical, vendors), and gender. Our eligibility criteria included providing senior-level leadership in the field of health informatics and affiliation with a leading clinical, academic, public, or vendor organization. Experts were identified through the research team’s international academic and professional networks.

#### Development and Piloting of Candidate Capabilities

Our focus was to ensure that the proposed list of candidate technological capabilities forming the basis of the eDelphi exercise drew on ongoing work relating to digital excellence in hospitals. We used NHS England’s Digital Maturity Index as a basis for constructing the initial list [9]. The index was developed in 2013 based on HIMSS EMRAM but included additional dimensions of interoperability, technological readiness, and infrastructure. We then piloted this initial list with three clinical academics, which resulted in some changes to the wording to improve clarity.

#### Round 1 of the eDelphi

Identified experts received an invitation email explaining the rationale for and aim of the study, the reason they were invited, what taking part would involve, and a personalized link to the Round 1 online survey. Experts were asked to follow the link if they wished to participate. We sent up to three follow-up emails at 2-3 week intervals to those who did not complete the survey following the initial invitation.

The opening page of the online survey for Round 1 contained further details of the study and a link to a participant information sheet. We obtained informed consent from each participant before the start of the survey. Participants were given the option to receive a summary of the findings once the study was completed. The main body of the online survey consisted of the list of proposed technological capabilities identified in the piloting stage. Participants were asked to rate how much they agreed that each proposed capability could be used to assess the level of digital excellence in hospitals, using a scale ranging from 1 (strongly agree) to 9 (strongly disagree). Experts were also encouraged to comment on each capability to suggest more appropriate wording, merge, split, or remove the capability, or to add other comments. Finally, we asked experts for suggestions of any additional capabilities they wished to add to the list.

#### Analysis of Data From Round 1

The purpose of analysis at this stage was to produce material for Round 2 of the eDelphi exercise. First, we revised the list of proposed capabilities based on participants’ comments from Round 1, changing the wording and dividing some capabilities into two or more capabilities to improve precision and clarity. We also added capabilities proposed in Round 1 to the revised list. As the majority of candidate capabilities were revised following insights from Round 1, we decided not to remove any capabilities at this stage. We further produced a feedback document that contained a summary of experts’ comments and descriptive statistics from Round 1 for each capability.

#### Round 2 of the eDelphi

Experts who completed Round 1 were invited to take part in Round 2 via an invitation email as before. Again, we sent up to three reminders at 2-3 week intervals to those who did not complete Round 2 following the initial email. An online version of the feedback document from Round 1 was also provided. Experts were given their score for each revised capability and asked if they wished to reconsider given the feedback from Round 1. If they replied “Yes,” they were given an option to amend their assessment using the same scale as in Round 1. Experts were asked to rate how much they agreed that each proposed new capability could be used to assess the level of digital excellence in hospitals on the same scale as for other capabilities. Experts were also able to comment on each capability, as above.

#### Analysis of Data From Round 2 and Definition of Consensus

Analysis following Round 2 aimed to identify any consensus on the capabilities and determine whether an additional round was needed. We defined consensus a priori as 70% agreement among experts that a specific capability should be included [17]. In other words, to be included, at least 70% of experts needed to “agree” or “strongly agree” to the appropriateness of the capability to define digital excellence in hospitals. After calculating the percentage of experts agreeing or strongly agreeing that the capability should be included, we removed all capabilities for which fewer than 70% agreed (see Multimedia Appendix 1), to produce the final list of capabilities.

#### Qualitative Data Collection and Analysis

To supplement the consensus-building exercise with additional insights, we incorporated several open-ended questions into the surveys, for which experts were able to provide free-text responses. In Round 1, we gave one current definition of digital maturity proposed by the MIT Sloan Management Review and asked experts to comment on this definition in the health care context [22]. We also asked experts to comment on the role of nontechnological factors (eg, strategy, workforce, culture) in the context of digital excellence in hospitals. Some feedback from Round 1 suggested that the initial list of capabilities focused too narrowly on the internal operations of hospitals. In Round 2, we therefore asked experts to comment on (a) conceptualization of digital excellence in hospitals in the context of the broader health care ecosystem; and (b) digital excellence in the context of a patient-centered health care perspective. We analyzed data from all free-text entries using thematic analysis to identify key themes of digital excellence in health care [23]. The qualitative data were initially analyzed by one researcher (MK). The resulting coding framework and the analyses were then reviewed and expanded by the wider team (KC, RW, AS). Researchers from a variety of backgrounds (eg, social sciences, public health, informatics) were involved in the analysis of the qualitative data, and diverging findings and viewpoints were discussed in detail in order to minimize the risk of bias.

## Results

### eDelphi Process and Expert Characteristics

**Figure 1:**
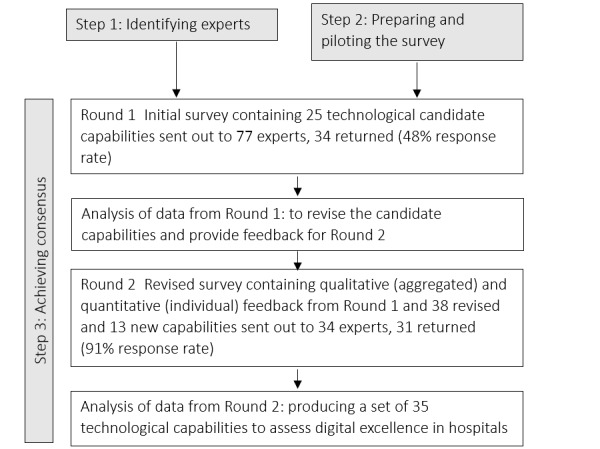
Flow diagram for the eDelphi exercise.

In total, 77 experts were invited. Of these, 34 agreed to take part and completed Round 1 (44% response rate), and 31 of the 34 completed Round 2 (91% response rate). Table 1 describes the characteristics of the 31 experts who took part in both rounds; Figure 1 outlines the steps involved.

**Table 1:** Expert characteristics.

Characteristic				Experts approached to take part (n=77), n (%)		Experts who took part in both rounds (n=31), n (%)	
**Sector**							
	Clinical			17 (22)		6 (19)	
	Academia			35 (45)		17 (55)	
	Policy			5 (6)		1 (3)	
	Vendor			20 (26)		7 (23)	
**Region**							
	**North America**			21 (27)		12 (39)	
			United States		21 (27)		12 (39)
	**South America**			2 (3)		1 (3)	
			Brazil		2 (3)		1 (3)
	**Europe**			46 (60)		15 (48)	
			United Kingdom		23 (30)		6 (19)
			Spain		6 (8)		4 (13)
			Norway		4 (5)		2 (7)
			Denmark		3 (4)		1 (3)
			Sweden		2 (3)		1 (3)
			Slovenia		2 (3)		1 (3)
			Belgium		1 (1.5)		0 (0)
			Estonia		1 (1.5)		0 (0)
			Finland		1 (1.5)		0 (0)
			Russia		1 (1.5)		0 (0)
			Austria		1 (1.5)		0 (0)
			Germany		1 (1.5)		0 (0)
	**Australasia**			6 (8)		3 (10)	
			Australia		6 (8)		3 (10)
	**Middle East**			2 (3)		0 (0)	
		Saudi Arabia		1 (1.5)		0 (0)	
		Israel		1 (1.5)		0 (0)	
**Gender**							
	Female			11 (14)		4 (13)	
	Male			66 (86)		27 (87)	

### Digital Excellence in Hospitals

Experts identified 35 technological capabilities that were judged to characterize digital excellence in hospitals (Tables 2-4). The technological capabilities fell into two categories: (a) capabilities within a hospital, and (b) communication with other parts of the health care system, and with patients and carers. The need to distinguish between capabilities within hospitals and those relating to the broader context of the health care ecosystem was emphasized in free-text comments, for example:

There is an important assessment on where enterprises (eg, hospitals) are, versus where those enterprises sit in an ecosystem and how they interact with those wider ecosystems.

Vendor

#### Technological Capabilities Within a Hospital

The largest category, technological capabilities within a hospital, included 20 items (Table 2), including technologies to promote the appropriate use and administration of medication, capabilities to capture structured and unstructured data, and the ability to integrate new advanced technologies (eg, natural language processing) within existing systems.

The most substantial proportion of capabilities within this category (five of 20) related to medicines management. The highest level of agreement was applied to a capability related to closed-loop electronic medicines management stating that it should be included as a marker of digital excellence (90% agreement), this was closely followed by capabilities relating to the effective capture of clinical data.

Experts proposed four new capabilities in Round 1 (Capabilities 10, 12, 13, and 15; Table 2). These were concerned with advancements in electronic medical records, electronic prescribing and medicines administration systems to improve user experience (for example Capability 10 ‘A single list of all medication for one patient is available’), and integration of new technologies and analytical approaches into existing systems (for example Capability 15 ‘Use of machine learning and adding third party programs through Application Programming Interfaces’).

**Table 2:** Technological capabilities within hospitals.

Agreed list of capabilities	“Strongly agreed” and “agreed”^a^ (%)	Number of experts who agreed (n=31)	Median	IQR^b^
1. Closed-loop electronic medicines management and optimization (electronic prescribing with technology-assisted identification of both patient and medication, eg, bar codes or RFID^c^ tags)	90	28	1	1-2
2. Effective mechanisms to collect and record complete, accurate and high-quality patient/clinical data	87	27	2	1-2
3. Structured data (records, assessments, and plans) captured digitally at the point of care	87	27	1	1-2
4. Orders (eg, lab tests) are ordered, and results reported in a coded form (ie, using standard compendiums and international vocabulary standards, including dm+d^d^, and acknowledged electronically in the system	84	26	1	1-2
5. Effective mechanisms to review and improve the quality of patient/clinical data	84	26	2	1-2
6. Flexible digital systems guiding clinicians along evidence-based, person-specific, clinical pathways	81	25	2	1-2
7. Unstructured data (eg, notes, free text) captured at the point of care when appropriate	81	25	2	1-2
8. Person reading/acting on the results acknowledges this electronically in the system	81	25	1	1-2
9. Cybersecurity strategy and continuity processes in place and implemented effectively	81	25	1	1-2
10. A single list of all medication for one patient is available^e^	81	25	1	1-2
11. Management intelligence through digital health data	81	25	1.5	1-2
12. Reducing the need for duplicate entry of patient data to near-zero^e^	81	25	2	1-2
13. Third-party tools can be added through Application Programming Interfaces^e^	81	25	2	1-2
14. Advanced clinical decision support (eg, integrated with lab data, diagnosis codes) with alerts that are both sensitive and specific and therefore less likely to result in alert fatigue	77	24	2	1-2
15. Use of machine learning and automation when appropriate (eg, analysis of radiology images)^e^	77	24	2	1-2
16. Clinical intelligence through digital health data	77	24	1	1-2
17. The ability to monitor outcome data for modifying clinical pathways based on digital tools and services	77	24	2	1-2
18. Open Application Programming Interfaces allowing different software components to interact	74	23	1	1-3
19. Supporting end-to-end redesign and improvement of clinical pathways based on digital tools and services	74	23	2	1-3
20. Advanced analytics capability to support the move from reactive to proactive/predictive models of care	74	23	2	1-3

^a^Experts scored each capability using a scale ranging from “1” (strongly agree) to “9” (strongly disagree).

^b^IQR: Interquartile range.

^c^RFID: Radio Frequency Identification.

^d^dm+d: Dictionary of Medicines and Devices.

^e^New capabilities suggested by experts in Round 1 of the eDelphi.

#### Communication With Other Parts of the Health Care System, Patients, and Carers

This category was related to enabling the exchange of information and communication beyond an individual hospital setting, including communication with other parts of health and social care systems (Table 3), and communication with patients and carers (Table 4). In total, this category comprised 15 capabilities, of which ten related to communication with other parts of health care systems and five to communication with patients and carers. Experts proposed two new capabilities, including the use of a unique patient identifier (Capability 23, Table 3), and the ability to exchange information with other systems based on shared standards (Capability 6, Table 3).

**Table 3:** Technological capabilities related to communication with other parts of the health and social care system.

Agreed list of capabilities	“Strongly agreed” and “agreed”^a^ (%)	Number of experts who agreed, (n=31)	Median	IQR^b^
1. Exchange of prescription information in a structured way within and between organizations and sectors	87	27	1	1-2
2. Local sharing of relevant data across the local health care ecosystem facilitated by interfacing or interoperability of electronic systems	84	26	1	1-2
3. A unique patient identifier used across the health care system^c^	84	26	1	1-2
4. Data analysis at scale and use of insights to deliver targeted care for high-risk and high-use groups of patients (eg, diabetes, chronic obstructive pulmonary disease, asthma) across a population or area	84	26	2	1-2
5. Using digital systems to enable the seamless (through interfaces/integration) flow and use of information/data across organizational boundaries within a local health care ecosystem	81	25	1	1-2
6. Ability to interoperate with other standard-based external systems^c^	81	25	2	1-2
7. Referrals within and between hospitals are always managed electronically	77	24	1	1-2
8. Ability to send communications to primary care and social care through a variety of media	77	24	2	1-2
9. Ability to produce data for audits and other reports based on the routine collection of complete, accurate, and quality data	74	23	2	1-3
10. Discharge to primary care and community is always managed electronically	71	22	1	1-2

^a^Experts rated how much they agree that the capability can be used to assess the level of digital excellence in hospitals on a scale from “1” (strongly agree) to “9” (strongly disagree).

^b^IQR: Interquartile range.

^c^New capabilities suggested by experts in Round 1 of the eDelphi.

**Table 4:** Technological capabilities related to communication with patients and carers.

Agreed list of capabilities	“Strongly agreed” and “agreed”^a^ (%)	Number of experts who agreed, (n=31)	Median	IQR^b^
1. Records, assessments, and plans shared digitally and easily accessible to patients and carers to enter and amend the data securely and confidentially	90	28	1	1-2
2. Records, assessments, and plans shared digitally and easily accessible to patients and carers to view the data securely and confidentially	87	27	1	1-2
3. Ability to receive communications from patients and carers through a variety of media	74	23	2	1-3
4. Ability to send communications to patients and carers through a variety of media	74	23	2	1-3
5. Using mobile technologies to support the delivery of care outside traditional settings and closer to home	71	22	2	1-3

^a^Experts rated how much they agree that the capability can be used to assess the level of digital excellence in hospitals on a scale from “1” (strongly agree) to “9” (strongly disagree).

^b^IQR: Interquartile range.

### Broader Aspects of Digitally Enabled Change: Culture, Skills, and Strategy

Free text responses emphasized that technologies should not be viewed in isolation and that social and organizational factors were crucial for digital transformation (Table 5).

Organizational culture, characterized by a willingness to transform established ways of working and an openness to risk-taking, was frequently mentioned as key to promoting digital transformation:

It is important to have a culture where individuals are prepared to change their ways of working and take some risks with an understanding of the overall good that will be achieved.

Policy expert

Experts also frequently mentioned the need for a diverse set of interdisciplinary skills supporting these transformations. Here, participants called for a range of technological, project management, and business expertise:

Digital health is a diverse, interdisciplinary sector, something that is reflected in the skills required in the field, ranging from higher-level computing, such as software development and software engineering to project management and business-related skills.

Vendor

Experts further highlighted the need for sufficient resources for the existing staff base, and their emerging training needs to support digital transformation:

A digital agenda cannot be delivered without sufficient staff, who are experienced and well trained within the digital team.

Clinician

**Table 5:** Social and organizational factors contributing to digital maturity.

Factor	Description
Organizational culture	Willingness to face the new, change the way of thinking, and to take risks Culture of allowing innovations Understanding of change management Culture free of bullying and harassment Leadership to support digital transformation
Workforce	Skills within the digital team: software development, software engineering, project management, business-related skills Skills across the hospital’s workforce: the ability to perform one’s role using digital tools Professionalization of health informatics
Strategy	Putting clinical benefits at the center of clinical strategy Aligning digital strategy with the overall strategy of the hospital Support of the digital agenda from the hospital’s board

## Discussion

### Summary of Findings

We have established consensus on a discrete set of internationally relevant technological capabilities to indicate digital excellence in hospitals. Engaging international experts in a transparent process represented by the Delphi technique, allowed us to develop a detailed, multi-axial mapping of digital excellence, which may be used by decision-makers to inform digital transformation strategy and evaluation. The outcomes of this eDelphi process mark a significant departure from existing tools such as HIMSS EMRAM and the NHS Digital Maturity Index [8,9]. First, our results point to a shift away from the description of purely technological functionalities towards digital transformation capabilities and highlight a need to be cognizant of cultural and strategic factors, such as skills and resources, to support the digitally enabled transformation of health care. Second, our findings indicate that the concept of digital excellence is moving beyond the physical boundaries of acute hospitals. Thus, once a certain level of digitization and data sharing is achieved within hospitals, strategic direction needs to shift towards sharing data and integration across local/regional/national ecosystems that encompass primary and social care providers and enable patient self-management.

### Strengths and Limitations

This study is the first attempt to achieve international consensus on a defined set of technological capabilities to indicate digital excellence in hospital settings. We recruited a relatively large sample of international experts from a variety of countries and achieved a good overall response rate. There is considerable variation in the number of experts involved in Delphi studies, and no consensus exists as to what constitutes an optimal number of experts [24,25]. However, the available evidence indicates that the Delphi technique produces reliable outcomes for sample sizes of 20 or above, and that increasing the number of experts above that number does not significantly change the outcomes [25]. We do, however, acknowledge that including a larger number of experts in this eDelphi could have provided valuable additional insights. Our participants reached consensus after two rounds, and we decided not to conduct further rounds. Evidence in the literature indicates that Delphi studies consisting of two to three rounds are preferred [17]. Additionally, in the case of busy experts and clinicians (as was the case for this work), response exhaustion occurs after two rounds resulting in limited new insights occurring in consequent rounds [26].

Our identified criteria have the potential to be used internationally, although our sampling reflects a certain subset of predominantly English-speaking economically developed countries and, therefore, high-performing health care systems. Our sample also exhibits a strong gender bias, with 27 of 31 Round 2 participants being men. This bias may mirror a broader gender bias present across the digital health leadership community but is likely to affect our findings and conclusions.

A more general concern is that the eDelphi process itself has some limitations. It may, to some extent, force consensus and reinforce dominant views (although controlled anonymized feedback should minimize normative pressure to align views) [16,27]. The addition of a qualitative component may have helped mitigate against this risk by allowing dissenting voices to be heard and by allowing discussion of the complexity of the context in which attempts to measure excellence are taking place. While we originally intended to examine differences between groups of experts (eg, experts from different regions and commercial and public sector) with regards to ratings of different capabilities, we have not been able to conduct those analyses meaningfully given the sample size and the small overall variance in our data.

### Integration of Findings With the Current Literature

Most existing models seeking to define digital excellence in health care settings are hospital-focused and stage-based [28]. Our findings question the appropriateness of such one-directional models, which assume that organizations and people within them progress towards increasingly advanced levels of maturity through a predefined set of consecutive stages associated with certain characteristics. Indeed, numerous accounts of organizations leapfrogging undermine the tacit idea of stage-based models going through a fixed sequence of stages [29]. Stage-based models are popular, perhaps because they promise a simple way to measure progress, but give little scope for health systems and individual organizations to articulate their local priorities [12]. Furthermore, ‘one size fits all’ assessment criteria enforce a common standard even under circumstances where achieving this may not be appropriate or impose disproportionate costs. Maturity models from outside the health sector can offer insights into possible ways of addressing some of the limitations of the currently dominant, linear, one-directional approaches in health care. For example, the Deloitte Maturity Model [30] developed primarily to meet the needs of the telecommunications industry, proposes to assess digital maturity using 179 digital capabilities grouped into five categories representing the core dimensions for the functioning of an organization (eg, ‘customers’, ‘operations’). An organization can choose which capabilities from which dimension to develop and in what order, based on its local priorities. This flexibility, in turn, allows for articulating local needs and following specific digital journey appropriate for the local context. Our findings support the increasing recognition that particular organizational and cultural environments of health systems are important factors when considering digital excellence [10,31].

The existing literature predominantly places large acute hospitals at the center of discussions of digital excellence [32]. Our study highlights how the entrenched focus on acute hospitals can draw attention away from integration across the health care ecosystem—even though integrated, patient-centered care has become a key component of current health policies internationally [33]. In line with this, HIMSS Analytics recently developed the Continuity of Care Maturity Model (CCMM) [34]. CCMM, like EMRAM, comprises seven stages and includes dimensions such as interoperability, exchange of information, coordination of care, patient involvement, and use of HIT to optimize clinical and financial outcomes. However, this extended HIMSS classification focuses on the individual health care provider rather than considering the entire health care system, and it remains a stage-based approach. It also remains mainly relevant to the hospital-centric United States context.

There is only limited evidence that meeting all criteria in any index of digital excellence leads to improved quality, safety, or efficiency outcomes, although some functionalities such as clinical decision support systems have been shown to improve practitioner performance [35,36]. At the same time renewing digital infrastructure to meet the ever-expanding requirements for each progressive stage of digital maturity indexes such as HIMSS EMRAM is costly. Many hospitals might choose not to pursue the route of advancing across stages of digital maturity due to high costs combined with insufficient evidence of desired return on investment. Thus, although digital excellence indices are commonly viewed as a proxy measure for improvement in efficiency and safety, there is limited evidence that adoption of these models will *per se* deliver such improvements [37].

### Implications for Research, Policy, and Practice

The identified technological capabilities have the potential to serve as a practical means to baseline and measure digital progress within acute hospital settings and their wider health care context. They can also promote international comparisons. Future work should focus on developing an assessment tool based on these identified capabilities. This needs to include establishing a scoring mechanism and weighting criteria for the capabilities comprising the tool and demonstrating the tool’s reliability and validity, including responsiveness to change and discriminatory properties. This work could be facilitated by using the set of capabilities to assess digital excellence in selected hospitals worldwide. It might also be valuable to investigate whether our set of capabilities could be further divided into more detailed categories to provide a better understanding of dimensions constituting digital excellence in hospitals. Additionally, there is a need for further efforts aiming to develop an agreement on what constitutes digital excellence for health care providers that includes views of additional stakeholders such as politicians, decision makers, and authorities.

### Conclusions

We have identified an internationally agreed defined set of technological capabilities that constitute digital excellence in hospitals. Our study also foregrounds the managerial and cultural skills necessary for successful, digitally enabled change. Finally, it highlights the need to address integrating digital capabilities across the wider health and care ecosystem to deliver safe, high-quality, and patient-centered care. Digital implementation strategies and indicators need to be positioned within this wider health system landscape to enable and foster transformational change in health care internationally.

## Appendix

Multimedia Appendix 1 Capabilities that did not meet the inclusion criteria.

## Abbreviations

CCMM: Continuity of Care Maturity Model
CPOE: Computerized Physician Order Entry
EMR: electronic medical record
EMRAN: Electronic Medical Record Adoption Model
HIMSS: Healthcare Information and Management System Society
HIT: health information technology
NHS: National Health Service
