# Supplementary material for: Technological Capabilities to Assess Digital Excellence in Hospitals in High Performing Health Care Systems: International eDelphi Exercise
Source: J Med Internet Res. 2020 Aug 18;22(8):e17022. doi: 10.2196/17022 (PMC7463397; doi:10.2196/17022)
Supplement: Multimedia Appendix 1 [file jmir_v22i8e17022_app1.docx]

**APPENDIX 1: CAPABILITIES THAT DID NOT MEET THE INCLUSION CRITERIA**

Almost half (seven of 15) of the capabilities that did not meet the consensus criteria were new capabilities proposed in Round 1. These capabilities related to technologies that were at an early stage of development (e.g. ‘*Capturing of clinicians’ thinking (diagnostic assessments, reasons for medications, reasons for overriding alerts, reasons for discontinuing medications)’*) and solutions requiring a high level of integration across the health and care system (e.g. *‘Link in with third sector, social care, emergency services’*).

Table 1 Capabilities for which there was no consensus for inclusion at the end of Round 2

| An agreed list of capabilities^a^ | Percentage ‘*Strongly agreed*’ and ‘*Agreed*’ | Number of experts agreed  (N=31) | Median | IQR^b^ |
| --- | --- | --- | --- | --- |
| 1. Clinical data recorded in a structured format when appropriate | 68 | 21 | 2 | 1-2 |
| 2. Effective tools available to code narrative data where appropriate | 68 | 21 | 2 | 1-2 |
| 3. Standard clinical terminology used for the direct management of care where appropriate | 68 | 21 | 2 | 1-2 |
| 4. Effective tools available to extract standard clinical terminology from natural language input | 68 | 21 | 2 | 1-2 |
| 5. Access to clinical knowledge management tools and services (e.g., ePrescribing Decision Support System (DSS), National Institute for Health and Care Excellence (NICE) / Scottish Intercollegiate Guidelines Network (SIGN) guidance, hospital antimicrobial handbook, lab handbook etc.)^c^ | 68 | 21 | 2 | 1-3 |
| 6. Digital technologies (e.g. apps & digital therapies) to help patients manage long-term conditions more effectively and take greater control of their own care | 68 | 21 | 2 | 1-2 |
| 7. Patients and carers can review and add clinical data (e.g., to say how they are feeling)^c^ | 68 | 21 | 2 | 2-3 |
| 8. Infrastructure and governance in place for remote consultation with patients in other setting | 61 | 19 | 2 | 1-2 |
| 9. eConsent system for use of blood / tissue / data for research^c^ | 61 | 19 | 2 | 2-3 |
| 10. Linkages with hospital pharmacy dispensing data^c^ | 58 | 18 | 2 | 2-1 |
| 11. Functionality delivered at scale across a whole organization, health ecosystem or provider chain | 58 | 18 | 2 | 1-2 |
| 12. Asset and resource optimization combining digital health data and data not immediately captured electronically (e.g., interviews with stakeholders) | 48 | 15 | 3 | 2-3 |
| 13. Capturing of clinicians’ thinking (diagnostic assessments, reasons for medications, reasons for overriding alerts, reasons for discontinuing medications)^c^ | 45 | 14 | 3 | 2-4 |
| 14. Link in with third sector, social care, emergency services including police^c^ | 45 | 14 | 3 | 2-4 |
| 15. Effective Natural Language Processing tools^c^ | 29 | 9 | 3 | 2-5 |

^a^Experts scored each capability using a scale ranging from ‘1’ (Strongly agree) to ‘9’ (Strongly disagree)
^b^Interquartile range (IQR) is a measure of variability in a set of results that indicates the amount of spread in scores in the middle 50% of answers
^c^New capabilities suggested by experts in Round 1 of the Delphi study

Source: own depiction
